# Effect of adrenergic agonists on coronary blood flow: a laboratory study in healthy volunteers

**DOI:** 10.14814/phy2.12806

**Published:** 2016-05-24

**Authors:** Alvaro F. Vargas Pelaez, Zhaohui Gao, Tariq A. Ahmad, Urs A. Leuenberger, David N. Proctor, Stephan R. Maman, Matthew D. Muller

**Affiliations:** ^1^ Penn State Heart and Vascular InstitutePenn State University College of MedicineHersheyPennsylvania; ^2^ Division of General Internal MedicinePenn State University College of MedicineHersheyPennsylvania; ^3^ Department of KinesiologyNoll LaboratoryThe Pennsylvania State UniversityUniversity ParkPennsylvania

**Keywords:** Blood pressure, coronary circulation, exercise, sympathetic nervous system, vascular resistance

## Abstract

Myocardial oxygen supply and demand mismatch is fundamental to the pathophysiology of ischemia and infarction. The sympathetic nervous system, through *α*‐adrenergic receptors and *β*‐adrenergic receptors, influences both myocardial oxygen supply and demand. In animal models, mechanistic studies have established that adrenergic receptors contribute to coronary vascular tone. The purpose of this laboratory study was to noninvasively quantify coronary responses to adrenergic receptor stimulation in humans. Fourteen healthy volunteers (11 men and 3 women) performed isometric handgrip exercise to fatigue followed by intravenous infusion of isoproterenol. A subset of individuals also received infusions of phenylephrine (*n* = 6), terbutaline (*n* = 10), and epinephrine (*n* = 4); all dosages were based on fat‐free mass and were infused slowly to achieve steady‐state. The left anterior descending coronary artery was visualized using Doppler echocardiography. Beat‐by‐beat heart rate (HR), blood pressure (BP), peak diastolic coronary velocity (CBVpeak), and coronary velocity time integral were calculated. Data are presented as M ± SD. Isometric handgrip elicited significant increases in BP, HR, and CBVpeak (from 23.3 ± 5.3 to 34.5 ± 9.9 cm/sec). Isoproterenol raised HR and CBVpeak (from 22.6 ± 4.8 to 43.9 ± 12.4 cm/sec). Terbutaline and epinephrine evoked coronary hyperemia whereas phenylephrine did not significantly alter CBVpeak. Different indices of coronary hyperemia (changes in CBVpeak and velocity time integral) were significantly correlated (*R* = 0.803). The current data indicate that coronary hyperemia occurs in healthy humans in response to isometric handgrip exercise and low‐dose, steady‐state infusions of isoproterenol, terbutaline, and epinephrine. The contribution of *β*1 versus *β*2 receptors to coronary hyperemia remains to be determined. In this echocardiographic study, we demonstrate that coronary blood flow increases when *β*‐adrenergic receptors are stimulated (i.e., during exercise and different intravenous infusions). Our infusion paradigms and beat‐by‐beat imaging methodologies can be used in future studies to evaluate age‐, sex‐, and disease‐ differences in adrenergic control of coronary blood flow.

## Introduction

A mismatch between myocardial oxygen supply and demand is fundamental to the pathophysiology of ischemia and infarction. In the coronary circulation, oxygen supply is primarily determined by oxygen content and coronary blood flow as the myocardium has very limited ability to enhance oxygen extraction (Duncker and Bache 2008; Heinonen et al. 2014). Coronary blood flow is controlled by metabolic, endothelial, and neural mechanisms, which are influenced by the sympathetic nervous system (Duncker and Bache 2008). In a similar way, the determinants of myocardial oxygen demand (i.e. wall stress, heart rate (HR), and contractility) are also heavily influenced by the sympathetic nervous system.

The relationship between the sympathetic nervous system and coronary blood flow is mediated by *α*‐adrenergic receptors (*α*‐ARs) and *β*‐adrenergic receptors (*β*‐ARs) (Feigl 1967; Barbato 2009). However, despite sophisticated experimentation in canine and porcine models (Miyashiro and Feigl 1993; Duncker et al. 1998; Tune et al. 2002; Duncker and Bache 2008; Gorman and Feigl 2012), the sympathetic control of coronary blood flow in healthy humans remains inadequately understood due to at least four important limitations. First, with respect to sympathetic control of coronary blood flow, dogs and pigs are quite different (Duncker and Bache 2008); thus species differences must be considered. Second, since the gold standard to measure coronary blood flow involves cardiac catheterization, most of the subjects in human studies have had a significant degree or suspicion of cardiovascular disease that indicated invasive testing; coronary artery disease clearly affects mechanical and autoregulatory flow responses (Mudge et al. 1976; Brown et al. 1984; Nabel et al. 1988; Hess et al. 1989; Zeiher et al. 1989; Vita et al. 1992). Third, the use of sedatives and opioids during cardiac catheterization are known to alter hemodynamic parameters such as HR, blood pressure (BP) and preload, as well as sympathetic and parasympathetic tone (Raza et al. 1989; Grossmann et al. 1996; Twersky et al. 2001). Fourth, to get a true assessment of coronary physiology in healthy humans the determinants of myocardial oxygen demand must be considered, preferably once the agonist infusions have elicited a steady state (i.e., not bolus injections)(Martinsson et al. 1989). Therefore, noninvasive experimental approaches (e.g., echocardiography studies in healthy subjects) that minimize these four limitations are likely to enhance our understanding of human coronary physiology.

The purpose of this study was to noninvasively quantify coronary responses to systemic adrenergic agonist infusions in healthy human volunteers under steady‐state conditions. Specifically, we used transthoracic Doppler echocardiography to measure coronary velocity responses in the distal left anterior descending artery. In separate protocols we infused four commonly prescribed, FDA‐approved medications known to alter the determinants of myocardial O2 supply and demand such as HR, contractility, BP, and coronary vascular resistance. We hypothesized that the *β*‐AR agonists isoproterenol, terbutaline, and epinephrine (and also isometric handgrip exercise) increase peak diastolic coronary blood velocity (CBVpeak) and that *α*‐AR agonist phenylephrine would decrease CBVpeak. Additionally, in order to address inconsistencies in the literature regarding coronary data analysis and reporting, we compared CBVpeak and coronary velocity time integral (VTI) responses.

## Materials and Methods

### Ethical approval

All study protocols were approved in advance by the Institutional Review Board of Penn State College of Medicine and conformed to the Declaration of Helsinki. All participants voluntarily provided written and informed consent.

### Design and subjects

These laboratory experiments used a repeated measures, within‐subjects design, and physiological variables were measured continuously during resting baseline, stressors (i.e., isometric handgrip or systemic infusions of adrenergic agonists), and recovery. All studies were conducted in the Clinical Research Center at Penn State College of Medicine with a physician (T. Ahmad or U. Leuenberger) physically present in the exam room during all agonist infusions.

Fourteen subjects (11 men and 3 women) volunteered for the study (Table 1). Prior to experimental visits, each subject underwent a screening visit. They received a standard history and physical examination, resting echocardiogram, dual‐energy X‐ray absorptiometry (DXA) scan, fasting blood panels (lipids and comprehensive metabolic panel), and finally a maximal treadmill exercise test with respiratory gas measurement (ParvoMedics) and 12‐lead EKG monitoring. A cardiologist interpreted all these tests prior to enrollment. The exclusion criteria were: pregnant or nursing women, those with resting heart rate below 45 bpm, history of cardiovascular, pulmonary, renal, or endocrine disease. All subjects reported being in good health and were asked to fast for 4 h, and avoid caffeine, alcohol, and exercise for 24 h before the studies.

**Table 1:** Baseline characteristics

	Mean ± SD	Minimum–Maximum
Age (year)	26 ± 16	22–67
Height (m)	1.77 ± 0.10	1.55–1.93
Weight (kg)	78.2 ± 14.5	55.6–101.9
Fat‐free mass (kg)	63.1 ± 13.0	40.8–81.0
Body mass index (kg/m^2^)	24.7 ± 2.8	20.0–30.4
Body fat (%)	19 ± 8	10–31
VO_2_ max (mL/kg/min)	44.3 ± 8.5	27–56

### Physiological measurements

All study protocols were conducted in the supine or left lateral position in a clinical research laboratory at 20–22°C. A three‐lead EKG (Cardiocap/5; GE Healthcare) to monitor HR was placed, as well as a finger BP cuff (Finometer, FMS), a pneumotrace to monitor respiratory movement, and an intravenous catheter in each arm. Prior to each stressor, three resting BPs were obtained by automated oscillometry of the right brachial artery (Philips Sure Signs VS3) after 15 min of quiet rest and these were used to verify the Finometer values as previously described (Muller et al. 2014b). All beat‐by‐beat variables were collected at 200 Hz by PowerLab (ADInstruments). CBVpeak and VTI in the distal left anterior descending coronary artery were obtained from the adjusted apical four‐chamber view using a GE Vivid 7 echocardiography system (all images acquired by Z. Gao). The specific procedures for measuring CBVpeak and in the LAD have been previously described by our laboratory (Momen et al. 2009; Ross et al. 2014). In brief, CBVpeak was calculated as the peak diastolic velocity (average of 3 or more cardiac cycles) and VTI was calculated as the area under the entire diastolic blood flow profile of the highest quality image for a single cardiac cycle. This approach is consistent with other published reports (Saraste et al. 2001; Meimoun et al. 2006; Abreu et al. 2014).

### Study protocol

After baseline measurements were obtained, all subjects performed isometric handgrip exercise at 40% of their maximal voluntary contraction until they reached fatigue. This maneuver is known to raise rate‐pressure product (RPP, the product of systolic BP and HR) (Gobel et al. 1978) and sympathetic nerve activity (Mark et al. 1985). Coronary flow data were collected throughout the trial. Rating of perceived exertion (RPE) was obtained after each handgrip exercise trial (6 = no exertion, and 20 = maximal exertion) (Borg 1982). All 14 subjects completed the handgrip trial.

**Figure 1:**
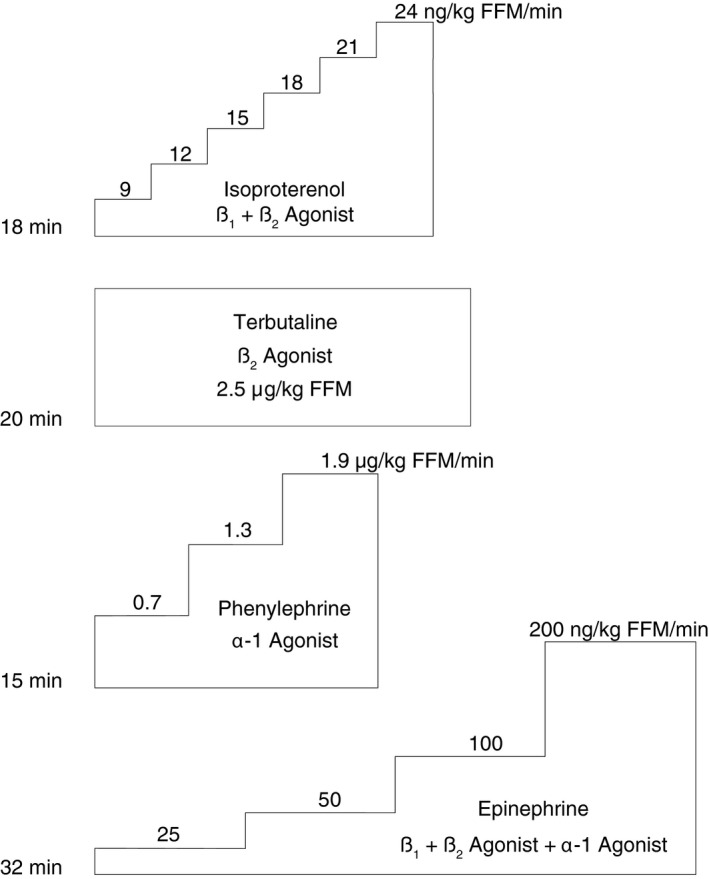
Experimental timeline for adrenergic agonist infusions. The times to the left signify the total duration of each infusion. FFM, fat‐free mass; please see text for details.

Figure 1 shows the four different infusion protocols. An infusion pump (Alaris PC Model 8015, CareFusion) was used to systemically infuse the agonists into a vein in the right arm; blood samples for glucose, potassium, and lymphocyte count were obtained from the left arm (these blood markers indicate the extent of *β*2 adrenergic stimulation) (Ahlborg and Ahlborg 1970; Kendall et al. 1982; Vincent et al. 1984; Van Tits et al. 1990) and samples were analyzed by Penn State Hershey Clinical Laboratories. The wash‐out period between infusions was at least five half‐lives in length and ranged between 15 and 30 min. Because terbutaline has a half‐life of 3–4 h, it was always performed last. All 14 subjects received isoproterenol, 10 received terbutaline, six received phenylephrine, and four received epinephrine.

All pharmacological agents were purchased from the Penn State Milton S. Hershey Medical Center pharmacy. Infusion dosages were calculated based on prior literature and clinical experience. Because total blood volume correlates with fat‐free mass (FFM) (Hunt et al. 1998), we chose to dose our drugs based on FFM instead of total body mass and we assumed that subjects in previous studies were 20% body fat. Prior to obtaining IRB approval, we performed simulations in Microsoft Excel to optimize each agonist infusion for a wide range of FFMs. This was an iterative process among team members and took into account the published literature, the functionality of the infusion pump, our desire to minimize the total volume of fluid given, and also our desire to minimize the number of procedural steps. In the end, we chose to prepare each agonist infusion identically for each subject and only the infusion rate was different between subjects (i.e., people with larger FFM received more mg of drug and also more volume per unit of time). For protocols in which the dose increased every stage, the infusion pump was stopped 10 sec before the end of the stage in order to enter the new dose. A detailed description of each adrenergic agonist is presented below along with its specific mixing instructions and infusion protocol.

*Isoproterenol hydrochloride* is a nonselective *β*‐AR agonist with an approximate half‐life of 2 min. It is supplied as 0.2 mg/mL ampule. For all studies, one mL of isoproterenol was added to 699 mL saline to form a solution with a concentration of 0.0002857 mg/mL. We used an 18 min infusion that increased every 3 min (six total stages). The first stage was 9 ng/kg FFM/min for 3 min and the dose increased by 3 ng every 3 min until a level of 24 ng/kg FFM/min. The dosages for isoproterenol are similar to previously described methods (Richards et al. 2010; Robinson et al. 2010, 2011). Subjects received a total of 40–85 mL during the 18 min infusion. We planned to terminate the infusion if HR rose by more than 50 bpm or if the BP fell more than 20 mmHg, or if symptoms occurred, but this threshold was never reached. Coronary data were collected during baseline and during the last 20 sec of each minute. In this report, we present the last 20 sec of each stage. Blood samples for glucose, potassium, and lymphocytes were obtained from the opposite arm before and after the infusion; these blood markers indicate the extent of *β*2 adrenergic stimulation (Ahlborg and Ahlborg 1970; Kendall et al. 1982; Vincent et al. 1984; Van Tits et al. 1990).

*Terbutaline sulfate* is a *β*‐AR agonist that is relatively selective for *β*2‐ARs with an approximate half‐life of 3–4 h. It is supplied as a 1 mg/1 mL vial. For all studies, 1 mL of terbutaline was mixed with 199 mL of normal saline to create a solution with a concentration of 0.005 mg/mL. We infused 2.5 *μ*g/kg FFM at a constant rate for 20 min to approximate the methods used by Kendall and colleagues (Kendall et al. 1982; Rolf Smith et al. 1983; Nuttall et al. 2003). In total subjects received 20–40 mL during the 20 min infusion. The same termination criteria used during isoproterenol infusion was implemented (*n* = 1 terminated terbutaline early). Coronary data were collected during baseline and during the last 20 sec of each minute. In this report, we present data from the 10th minute of the infusion and also the last 20 sec of the infusion. Blood samples for glucose, potassium, and lymphocytes were obtained from the opposite arm before and after the infusion.

*Phenylephrine hydrochloride* is a selective *α*1‐AR agonist that is a powerful systemic vasoconstrictor with an approximate half‐life of 5 min. It is supplied as 10 mg/1 mL. For all studies, 1 mL of phenylephrine was mixed with 399 mL of normal saline to produce a solution with a concentration of 0.025 mg/mL. We used a 15 min infusion and the dose increased every 5 min. The first stage was 0.7 *μ*g/kg FFM/min, followed by 1.3 *μ*g/kg FFM/min, and finally 1.9 *μ*g/kg FFM/min. These dosages are similar to previous reports (Davy et al. 1998; Cui et al. 2002). Subjects received a total of 35–60 mL during the 15‐min infusion. The infusion ended if the mean arterial BP increased by more than 25 mmHg or if HR fell by more than 15 bpm, or symptoms occured. Five of the six subjects reached these criteria in stage 3 so we chose to only report coronary data from stage 2.

*Epinephrine hydrochloride* is a sympathomimetic agent that acts predominantly on *β*‐ARs as well as *α*‐ARs (to a lesser extent) with an approximate half‐life of 2 min. It is supplied as 1 mg/mL vial. For all the studies, we mixed 1 mL of epinephrine with 499 mL of normal saline to produce a solution with a concentration of 0.002 mg/mL. We used a 32 min infusion paradigm with increments every 8 min. The first stage was 25 ng/kg FFM/min, the second stage was 50 ng/kg FFM/min, the third stage was 100 ng/kg FFM/min, and the fourth stage was 200 ng/kg FFM/min. These methods are based on previous studies by Leenen et al. (Leenen et al. 2005, 2007). The termination criteria for the infusion were the same as in the isoproterenol infusion (one participant terminated early so in this case the last 20 sec of infusion were used as the peak response). In total subjects received a range 41–120 mL during epinephrine infusion. Coronary data were collected during baseline and in the last 20 sec of each stage. In this report, we present hemodynamics and coronary data from the last 20 sec of the epinephrine infusion. Blood samples for glucose, potassium, and lymphocytes were obtained before and after the infusion from the opposite arm.

### Data collection and statistical analysis

All variables were monitored continuously and data were analyzed offline at specific time points. The coronary data were analyzed using ProSolv 3.0. Changes (Δ) in beat‐by‐beat HR, BP, RPP CBVpeak, and VTI in response to the stressors were calculated. Subsequently, indices of myocardial oxygen supply to demand were calculated as: ΔCBVpeak/ΔRPP*1000 and also as ΔVTI/ΔRPP*10000 as has been previously described (Monahan et al. 2013). Bivariate correlations were conducted to compare physiological parameters. All data are presented as mean ± SD in the text unless otherwise stated. *P*‐values <0.05 were considered statistically significant.

## Results

**Figure 2:**
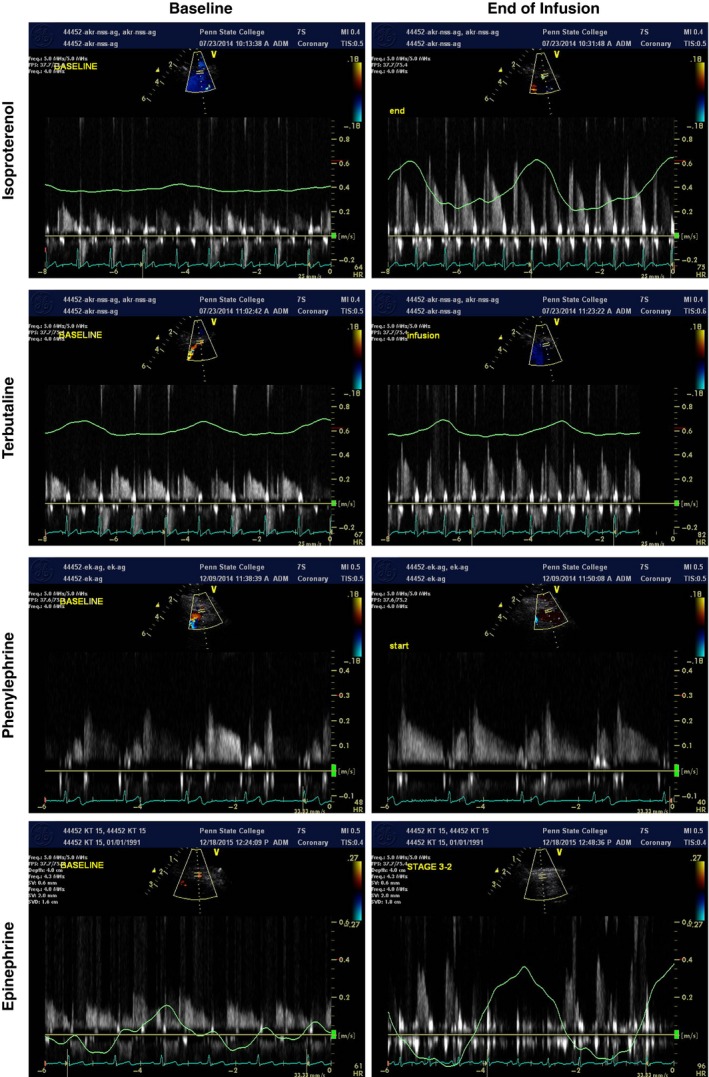
Representative echocardiographic images of coronary blood velocity (shown here in m/sec) obtained from the distal left anterior descending coronary artery during preinfusion baseline (left panels) and at the end of the agonist infusions (right panels). The peak of the diastolic velocity profile (CBVpeak) and the area under the curve (also called velocity time integral, VTI) were analyzed. The green waveform is the respiratory tracing. Please note the vertical scale is different for the phenylephrine recording.

Examples of coronary Doppler recordings are depicted in Figure 2.

### Handgrip

**Figure 3:**
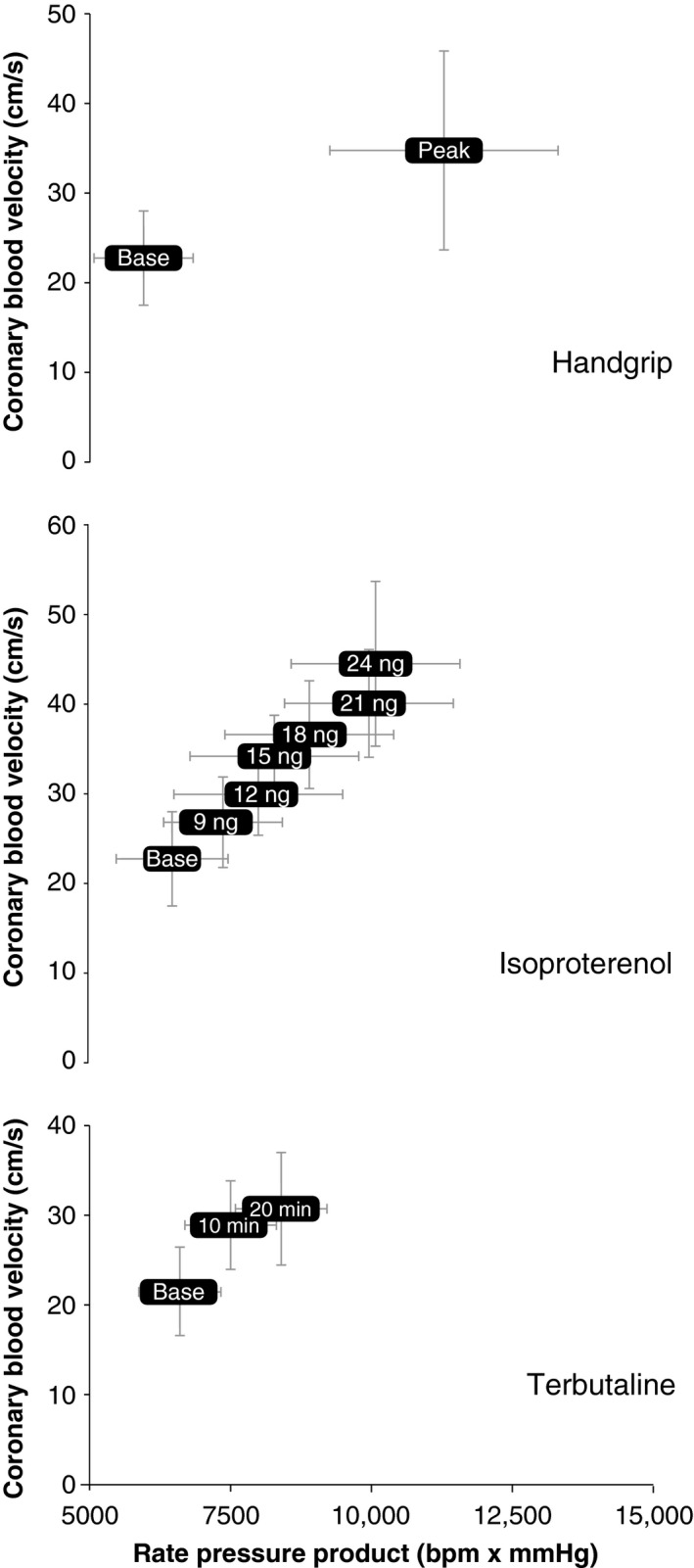
Peak coronary blood velocity and rate pressure product in response to different adrenergic stimuli. Error bars represent 2 standard deviations.

Isometric handgrip elicited significant increases in MAP, HR, RPP, CBVpeak, and VTI (Table 2 and Fig. 3).

**Table 2:** Hemodynamic and coronary responses

	Units	Baseline	Peak	*P*‐value
Handgrip				
* N* = 14				
* *MAP	mmHg	80 ± 7	117 ± 15*	<0.001
* *HR	bpm	56 ± 8	75 ± 9*	<0.001
* *RPP	bpm*mmHg	6117 ± 926	11349 ± 2075*	<0.001
* *CBVpeak	cm/sec	23.3 ± 5.3	34.5 ± 9.9*	<0.001
* *VTI	cm	9.6 ± 2.6	11.7 ± 3.1*	<0.001
Isoproterenol				
* N* = 14				
* *MAP	mmHg	83 ± 7	79 ± 6	0.093
* *HR	bpm	56 ± 10	77 ± 13*	<0.001
* *RPP	bpm*mmHg	6488 ± 1044	10067 ± 1673*	<0.001
* *CBVpeak	cm/sec	22.6 ± 4.8	43.9 ± 12.4*	<0.001
* *VTI	cm	8.6 ± 2.4	13.1 ± 3.8*	<0.001
Terbutaline				
* N* = 10				
* *MAP	mmHg	84 ± 7	81 ± 8*	0.014
* *HR	bpm	56 ± 8	69 ± 9*	0.001
* *RPP	bpm*mmHg	6570 ± 763	8373 ± 879*	<0.001
* *CBVpeak	cm/sec	20.9 ± 4.8	31.0 ± 6.6*	<0.001
* *VTI	cm	8.3 ± 2.0	10.5 ± 2.6*	0.012
Phenylephrine				
* N* = 6				
* *MAP	mmHg	86 ± 13	100 ± 15*	0.003
* *HR	bpm	55 ± 10	47 ± 6*	0.012
* *RPP	bpm*mmHg	6265 ± 781	6372 ± 1146	0.794
* *CBVpeak	cm/sec	23.0 ± 3.2	21.7 ± 6.2	0.438
* *VTI	cm	10.0 ± 1.2	10.8 ± 2.9	0.426
Epinephrine				
* N* = 4				
* *MAP	mmHg	83 ± 2	78 ± 4	0.360
* *HR	bpm	60 ± 10	79 ± 11*	0.001
* *RPP	bpm*mmHg	6805 ± 1042	10591 ± 1329*	0.016
* *CBVpeak	cm/sec	26.2 ± 5.3	39.1 ± 6.7*	0.001
* *VTI	cm	10.9 ± 3.0	11.6 ± 2.4	0.484

Hemodynamic and coronary responses to protocols that stimulate adrenergic receptors. Peak responses were obtained within the last 20 sec of the protocol. Mean arterial pressure (MAP), heart rate (HR) rate‐pressure product (RPP), peak diastolic coronary blood velocity (CBVpeak), velocity‐time integral (VTI). Data are shown as M ± SD; * indicates *P* < 0.05 compared to baseline.

### Isoproterenol

As expected, HR, RPP, CBVpeak, and VTI increased in response to 18 min of isoproterenol infusion (Table 2 and Fig. 3). By the end of the isoproterenol infusion MAP tended to be lower (approximately 4 mmHg decrease from baseline; *P* = 0.093) although this is unlikely to be clinically relevant. Our isoproterenol paradigm caused a small rise in blood glucose (from 77 ± 7 to 81 ± 7 mg/dL, *P* = 0.002) and a moderate increase in lymphocyte count (from 1.86 ± 0.38 to 2.37 ± 0.58 K/*μ*L, *P* < 0.001) but did not have a significant effect on potassium levels (from 4.1 ± 0.4 to 4.1 ± 0.4 mmol/L).

### Terbutaline

Terbutaline infusion for 20 min caused a slight decrease in MAP along with a rise in HR, RPP, CBVpeak, and VTI (Table 2 and Fig. 3). Our terbutaline paradigm caused a small rise in blood glucose (from 79 ± 7 to 85 ± 8 mg/dL, *P* = 0.001), a moderate reduction in potassium (from 4.0 ± 0.4 to 3.7 ± 0.5 mmol/L, *P* = 0.005), and a moderate increase in lymphocyte count (from 2.05 ± 0.43 to 2.68 ± 0.42 K/*μ*L, *P* < 0.001).

### Phenylephrine

Phenylephrine infusion increased MAP and decreased HR but had no significant effects on RPP, CBVpeak, or VTI (Table 2).

### Epinephrine

Epinephrine infusion increased HR, RPP, and CBVpeak but did not change VTI (Table 2).

### Comparison of CBVpeak to VTI

**Figure 4:**
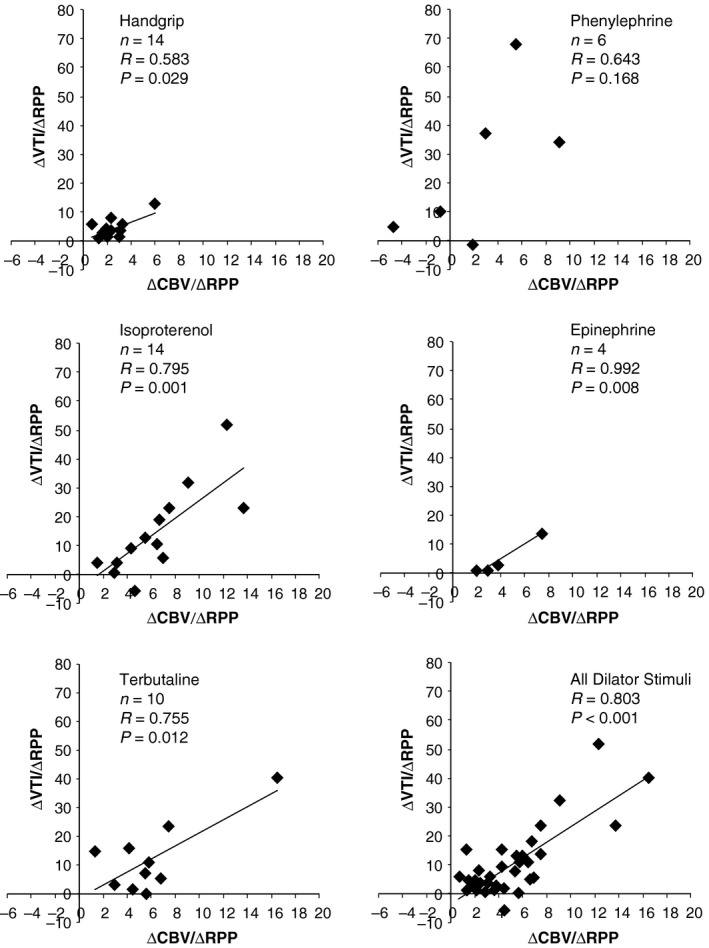
The derived variables ΔCBV/ΔRPP (*x*‐axes) and ΔVTI/ΔRPP (*y*‐axes) are both noninvasive indices of myocardial oxygen supply:demand balance. Correlations values are displayed for each adrenergic stimulus (data obtained during the last 20 sec of the protocols).

Figure 4 demonstrates that the changes in CBVpeak normalized to RPP were positively correlated with change in VTI normalized to RPP. We then compared percent changes in CBVpeak to percent changes in VTI and also found a positive correlation (*R* = 0.612, *P* < 0.001). It is worth noting that the magnitude of hyperemia (% change) was much less for VTI as compared to CBVpeak. Based on the regression line, a 50% increase in CBVpeak (due to an adrenergic vasodilator) resulted in only a 25% increase in VTI.

## Discussion

The purpose of this study was to noninvasively quantify coronary responses to systemic adrenergic agonist infusions in healthy human volunteers under rigorously controlled laboratory conditions. The current data were obtained using a heterogeneous sample of healthy men and women and indicate that coronary hyperemia occurs in response to low‐dose, steady‐state infusions of isoproterenol, terbutaline, and epinephrine (Figs 2 and 3). These responses were also noted when normalizing the hyperemic response to the metabolic stimulus (i.e., the ratio of coronary flow to RPP). Considering that different adrenergic agonist infusions had different effects on HR and BP (i.e., the primary determinants of myocardial oxygen demand), we believe normalizing coronary response to RPP is important. A secondary purpose of this study was to compare changes in CBV and VTI; the data in Figure 4 show these indices of myocardial oxygen supply are moderate to strongly correlated in response to *β*‐adrenergic receptor dilator stimuli. However, the responses to phenylephrine were inconsistent and further work is needed to determine the best index to quantify coronary blood flow noninvasively in humans.

The sympathetic nervous system plays an important role in myocardial oxygen supply and demand. The effects of sympathetic activation (e.g., during exercise, orthostasis, and thermal stress) are mediated through adrenergic receptors in the myocardium and coronary blood vessels. Prior data from our laboratory indicate that coronary hyperemia is observed during isometric handgrip exercise (Muller et al. 2011, 2012, 2014a; Ross et al. 2014) and cold pressor test (Monahan et al. 2013) in healthy humans, whereas coronary vasoconstriction occurs during simulated orthostatic stress (Gao et al. 2014), noxious cold stress to the forehead (Muller et al. 2014b), and inhalation of 100% oxygen (i.e., a nonsympathoexcitatory stimulus) (Gao et al. 2012, 2013). An alternative strategy to evaluate adrenergic receptor function in vivo is to infuse pharmacological agonists. To the best of our knowledge, this study is the first to evaluate coronary response to several different adrenergic agonists in humans. Because we infused the medications based on FFM (not total body mass) and measured coronary responses at time points when HR and BP were stable, we believe our methodology advances current knowledge.

As noted in the Introduction, prior studies performed in dogs, pigs, and humans undergoing cardiac catheterization cannot fully address healthy human physiology. Nevertheless, there is a large body of prior literature that warrants brief discussion. An early report by Gaal et al. (1966) demonstrated that intracoronary epinephrine increased coronary blood flow in anesthetized dogs; further studies showed that epinephrine infusion following pretreatment with propranolol evoked coronary vasoconstriction (Nayler et al. 1967). Several studies performed in the cardiac catheterization laboratory have also demonstrated that blocking *β*‐receptors with propranolol promotes vasoconstriction in response to epinephrine or sympathetic stress (Kern et al. 1983). Isoproterenol infusion in anesthetized dogs reduced coronary vascular resistance and the authors provided strong evidence that both *β*1 and *β*2 vascular receptors participated in this hyperemic response (Vatner et al. 1982). Whether *β*1 receptors play an important role in the coronary vasculature of humans (i.e., beyond the effects of myocardial and SA node *β*1 receptors affecting HR and contractility) remain unknown but *β*1 vascular receptors have been identified in a number of species, including humans (Ghaleh et al. 1995). The overwhelming majority of human studies have focused on *β*2‐mediated vasodilation (Sun et al. 2002; Barbato et al. 2005; Puri et al. 2012) and the consensus is that coronary artery disease impairs *β*‐mediated vasodilation and enhances *α*‐mediated vasoconstriction (Mudge et al. 1976; Brown et al. 1984; Nabel et al. 1988; Hess et al. 1989; Zeiher et al. 1989; Vita et al. 1992). Undoubtedly, peak coronary vasodilator responses to physiological and pharmacological challenges have prognostic value (Schachinger et al. 2000; Cortigiani et al. 2012). However, whether coronary adrenergic receptors play a major or minor role in maximal vasodilator responses (e.g., in response adenosine or dipyridamole) remains an unanswered question (Hodgson et al. 1989; Meimoun et al. 2006).

Previous echocardiography studies have used either CBVpeak (Meimoun et al. 2006; Abreu et al. 2014) or VTI (Chammas et al. 2007) to estimate change in coronary blood flow. The main difference between these two measures is that VTI takes into account diastolic time, a critical determinant of coronary blood flow (Heusch 2008), whereas CBVpeak does not and may therefore overestimate flow at increasing HR. Some studies have reported both CBVpeak and VTI (Kenny et al. 1994; Hildick‐Smith et al. 2000; Saraste et al. 2001; Lee et al. 2003) but did not provide detailed hemodynamic data (e.g., what was the HR, BP, and RPP when coronary measurements were obtained). Because of these inconsistencies in the published literature, we chose to compare CBVpeak and VTI during all stressors. Our correlation data in Figure 4 suggest that change in CBVpeak and VTI are similar during most stimuli that activate adrenergic receptors. However, phenylephrine infusion led to widely variable responses, likely because this stimulus directly increases coronary vascular resistance and also evokes baroreflex‐mediated bradycardia. Additional correlation analysis showed that percent changes in CBVpeak correlate to percent changes in VTI although the magnitude of vasodilation using VTI responses were only half as large as those using CBVpeak. The use of VTI has a theoretical advantage in echocardiographic analysis because it accounts for changes in the diastolic period; however, the measurement of VTI is more time consuming and is more image‐quality dependent. Taken together, our correlation data in Figure 4 are hypothesis‐generating and more definitive studies are needed to compare noninvasive coronary measurements of velocity to direct measurements of coronary blood flow.

The management of acute coronary syndromes and the success of defibrillation depend on optimizing and maintaining coronary perfusion during times when it is severely compromised. The advanced cardiac life support (ACLS) protocol is constantly evolving (Lavonas et al. 2015) although epinephrine and vasopressin continue to be mainstays of resuscitation. The current investigation was not designed to evaluate clinical guidelines but we speculate that epinephrine and other predominately *β*‐AR agonists play an important role in augmenting coronary perfusion which (with adequate chest compressions) ultimately increases the success of defibrillation in restoring spontaneous cardiopulmonary circulation. Additionally, our data suggest that phenylephrine is a suboptimal choice for treating hypotension (especially in patients with impaired myocardial perfusion due to coronary atherosclerosis or structural heart disease). Until more definitive clinical data are obtained, the debate over which vasopressor is best will continue (Egi et al. 2007; Morelli et al. 2008).

### Study limitations

Several factors may affect data interpretation. First, in order to calculate coronary blood flow, vessel diameter must be measured along with velocity. It has been documented that the percent increase in CBVpeak measured with transthoracic echocardiography is similar to the percent increase in coronary velocity measured by intracoronary Doppler guidewire (Momen et al. 2008) and that intracoronary Doppler guidewire measurements of percent increases in velocity correlated with percent increases in coronary blood flow (Reis et al. 1999). Most importantly, any detectable increases in LAD diameter due to vasoactive infusions are ~10 fold lower than changes in LAD blood velocity (Kiviniemi et al. 2007). Therefore, we believe our experimental approach is justified and changes in echocardiography‐derived coronary measurements are primarily due to changes in microvascular resistance rather than increases LAD diameter (or area).

Second, RPP is commonly used as a noninvasive index of myocardial oxygen consumption and it correlates strongly under resting conditions (Gobel et al. 1978). However, changes in myocardial contractility, as induced by *β*1 agonists, may alter this relationship. Studies have suggested that myocardial ischemia is influenced more by HR than BP (Loeb et al. 1978). Shorter diastolic periods observed at higher heart rates lead to less time for myocardial perfusion. Using vasodilator drugs can lead to reflex tachycardia, thereby worsening myocardial ischemia.

Third, it has been shown that the activation of *β*2‐receptors leads to endothelial release of nitric oxide, which induces vasodilation (Puri et al. 2012). Changes in coronary shear stress due to changes in HR and BP also contribute to the coronary hyperemia we observed. In addition, although nearly 85% of *β*‐adrenergic receptors found in coronary circulation belong to the *β*2 subtype, a small fraction of receptors are *β*3‐receptors, which contribute to nitric oxide‐mediated vasodilation (Dessy et al. 2004). Therefore, it is possible that some pharmacological agents believed to be specific for *β*1 and *β*2‐receptors also interact with *β*3 receptors and/or endothelial cells, thereby enhancing coronary vasodilation.

Fourth, phenylephrine causes an acute pressor response and reflex bradycardia, which lengthens the duration of diastole. From a mechanical perspective, these responses would be expected to have different effects on CBVpeak and VTI. The net effect of phenylephrine on coronary blood flow is challenging to predict and in our study RPP during the end of the infusion was not significantly different from baseline. Future work is needed to resolve this issue.

Fifth, in order to ensure maximal safety for our subjects, we chose to infuse the agonists slowly and in low dosages. It is likely that our doses caused submaximal vasodilation (compared to prior vasodilator studies with adenosine). Indeed, the blood markers we measured demonstrated only minimal changes compared to previous studies (Ahlborg and Ahlborg 1970; Kendall et al. 1982; Vincent et al. 1984; Van Tits et al. 1990).

Finally, dobutamine is often used to increase HR clinically and it has *α*1, *β*1, and *β*2‐AR affinity (Ruffolo 1987). We chose not to use dobutamine in this study because it is nonselective (i.e., the dose of dobutamine required to elicit an increase in HR would likely result in interaction with all adrenergic receptors). The control of coronary blood flow during exercise is particularly important to understand (Tune et al. 2002; Rowell 2004) and we believe future studies could isolate how *β*1 versus *β*2 vascular receptors participate in coronary exercise hyperemia in humans.

## Conclusions

To date, the understanding of adrenergic control of coronary blood flow has mostly come from experiments in dogs, pigs, and human patients with significant atherosclerosis (Duncker and Bache 2008). This study in healthy humans confirms and extends upon these prior investigations. Indeed, we show that coronary hyperemia occurs in response to isometric handgrip exercise as well as low‐dose, steady‐state infusions of isoproterenol, terbutaline, and epinephrine (Figs 2 and 3). The current methodologies can be used in future studies to evaluate age‐, sex‐, and disease‐ differences in adrenergic control of coronary blood flow.

## Conflict of Interest

None declared.
